# Tumor necrosis factor α-induced adipose-related protein expression in experimental arthritis and in rheumatoid arthritis

**DOI:** 10.1186/ar2779

**Published:** 2009-08-06

**Authors:** Asuka Inoue, Isao Matsumoto, Yoko Tanaka, Keiichi Iwanami, Akihiro Kanamori, Naoyuki Ochiai, Daisuke Goto, Satoshi Ito, Takayuki Sumida

**Affiliations:** 1 Division of Clinical Immunology, Advanced Biomedical Applications, Graduate School of Comprehensive Human Sciences, University of Tsukuba, 1-1-1 Tennodai, Tsukuba 305-8575, Japan; 2 PRESTO, Japan Science and Technology Agency, 4-1-8 Honcho Kawaguchi, Saitama 332-0012, Japan; 3 Department of Orthopedic Surgery, Advanced Biomedical Applications, Graduate School of Comprehensive Human Sciences, University of Tsukuba, 1-1-1 Tennodai, Tsukuba 305-8575, Japan

## Abstract

**Introduction:**

Tumor necrosis factor-alpha (TNFα) plays a pivotal role in rheumatoid arthritis (RA); however, the mechanism of action of TNFα antagonists in RA is poorly defined. Immunization of DBA/1 mice with glucose-6-phosphate isomerase (GPI) induces severe acute arthritis. This arthritis can be controlled by TNFα antagonists, suggesting similar etiology to RA. In this study, we explored TNFα-related mechanisms of arthritis.

**Methods:**

First, we performed GeneChip analysis using splenocytes of mice with GPI-induced arthritis. Expression of TNFα-induced adipose-related protein (TIARP) mRNA and protein in spleens, joints and lymph nodes was evaluated, and fluctuation of TIARP mRNA was analyzed after administration of anti-TNFα monoclonal antibody (mAb). Localization of TIARP in spleen and joints was also explored. Six-transmembrane epithelial antigen of the prostate (STEAP) families of proteins, the human ortholog of TIARP gene, were also evaluated in human peripheral blood mononucleocytes and synovium.

**Results:**

Among the arrayed TNFα-related genes, the expression of TIARP mRNA was the highest (more than 20 times the control). TIARP mRNA was detected specifically in joints and spleens of arthritic mice, and their levels in the synovia correlated with severity of joint swelling. Treatment with anti-TNF mAb significantly reduced TIARP mRNA expression in splenocytes. Among the splenocytes, CD11b^+ ^cells were the main source of TIARP mRNA. Immunohistochemistry showed that TIARP protein was mainly localized in hyperplastic synovium. Among the STEAP family of proteins, STEAP4 was highly upregulated in joints of patients with RA and especially co-localized with CD68^+ ^macrophages.

**Conclusions:**

The results shed light on the new mechanism of action of TNFα antagonists in autoimmune arthritis, suggesting that TIARP plays an important role in inflammatory arthritis, through the regulation of inflammatory cytokines.

## Introduction

Rheumatoid arthritis (RA) is a chronic inflammatory disorder with a variable disease outcome and is characterized by inflammation of multiple joints. The prognosis of RA patients has improved significantly in recent years after the introduction of tumor necrosis factor-alpha (TNFα)-based therapy [1]. Despite the wide use of these biologics, their precise mechanisms of action in RA remain unclear.

Several animal models of RA have been described; however, the therapeutic benefits of TNF antagonists have been confirmed in only a few of these models. Schubert and colleagues [2] reported that continuous injections of human TNF receptor (TNFR) p75-IgG-Fc fusion protein (Etanercept) from days 0 to 9 completely protected against the development of arthritis in glucose-6-phosphate isomerase (GPI)-induced arthritis. In this regard, we recently demonstrated a clear therapeutic effect of anti-TNF monoclonal antibody (mAb) in mice with GPI-induced arthritis, and the therapeutic response correlated with the *in vitro *regulation of TNF production [3]. We also identified that anti-interleukin-6 (IL-6) receptor mAb blocks the development of GPI-induced arthritis [3,4]. These results indicate that the GPI-induced arthritis model is suitable for studying the mechanisms of action of TNFα antagonists as well as IL-6 antagonists in RA patients.

Using such a TNFα-dependent arthritis model, we investigated TNFα-related molecules by GeneChip analysis. The expression of TNFα-induced adipose-related protein (TIARP) was the highest in GeneChip study. TIARP was identified as a transmembrane protein that is highly regulated by TNFα in adipocytes [5]. Not only TNFα but also IL-6 regulated the expression of TIARP [6], suggesting the involvement of the inflammatory cascade in RA. To our knowledge, however, no information on its role in arthritis or its localization in joints has been published.

To explore the role of TIARP in arthritis, we conducted the present study in GPI-induced arthritis. TIARP mRNA and proteins were upregulated in joints and spleens in mice with GPI-induced arthritis. Administration of anti-TNFα mAb reduced TIARP mRNA in splenocytes. In arthritic mice, TIARP mRNA was expressed mainly in CD11b^+ ^cells in the spleen, and TIARP mRNA level was increased in the joints (accompanied by joint swelling), especially in hyperplastic synovium. Overexpression of the human TIARP counterpart, such as six-transmembrane epithelial antigen of the prostate-4 (STEAP4), was noted in the synovia of patients with RA. The results provide the first characterization of the role of TIARP in inflammatory arthritis.

## Materials and methods

### Glucose-6-phosphate isomerase-induced arthritis

Male DBA/1 mice (6 to 8 weeks old) were obtained from Charles River Laboratories (Yokohama, Japan). Recombinant human GPI was prepared as described previously [7]. Mice were immunized by intradermal injection of 300 μg of recombinant human GPI-GST (glutathione S-transferase) (hGPI) in emulsified complete Freund's adjuvant (CFA) (Difco Laboratories Inc., now part of Becton Dickinson and Company, Franklin Lakes, NJ, USA). Control mice were immunized with 100 μg of GST in CFA. Arthritic animals were assessed visually, and changes in each paw were scored on a scale of 0 to 3. A score of 0 indicates no evidence of inflammation, 1 indicates subtle inflammation or localized edema, 2 indicates swelling that is easily identified but localized to the dorsal or ventral surface of paws, and 3 indicates swelling on all aspects of paws, and the maximum possible score was 12 per mouse. The experimental protocol was approved by the Ethics Review Committee for Animal Experimentation of the University of Tsukuba (Japan).

### GeneChip analysis of splenocytes from glucose-6-phosphate isomerase-induced arthritis

The spleens of three GPI-GST (molecular weight [MW] = 89 kDa) (300 μg)-immunized DBA/1 mice were harvested on day 10. As a control, the spleens of three GST (MW = 26 kDa) (100 μg)-immunized DBA/1 mice were used. Total RNA was extracted from the splenocytes using ISOGEN (Nippon Gene Co., Ltd., Toyama, Japan), and then 15 μg of RNA was used for cDNA synthesis by reverse transcription followed by synthesis of biotinylated cRNA through *in vitro *transcription. After cRNA fragmentation, hybridization with mouse 430A2.0 GeneChip (Affymetrix, Santa Clara, CA, USA) with probes for 43,000 mouse gene ESTs (expressed sequence tags) was performed in accordance with the protocol provided by the manufacturer. Analysis was performed by gene expression software.

### Analysis of TIARP and tumor necrosis factor-alpha gene expression

Spleens and lymph nodes were isolated, cut into small pieces, and passed through cell strainers (BD Biosciences, Erembodegem, Belgium) to obtain single-cell suspensions. The remaining cells were washed twice with phosphate-buffered saline (PBS). Synovial tissues from the ankle joints were isolated and minced by scissors. Total RNA was extracted with ISOGEN in accordance with the instructions provided by the manufacturer. cDNA was obtained by reverse transcription with a commercially available kit (Fermentas, Glen Burnie, MD, USA). Primers sequenced were as follows: TIARP sense 5'-AGCCCACGTGGTCAAAGCAT-3' and antisense 5'-CCTTGGTCCAGTGGGGTGA-3' and glyceraldehydes-3-phosphate dehydrogenase (GAPDH) sense 5'-CGTCCCGTAGACAAAATGGT-3' and antisense 5'-GAATTTGCCGTGAGTGGAGT-3'.

All polymerase chain reactions (PCRs) were performed in a Takara PCR Thermal Cycler (Takara Bio Inc., Shiga, Japan). After denaturation at 95°C for 5 minutes, cycles were set at 10 seconds at 94°C, 10 seconds at 60°C, and 30 seconds at 72°C. Cycling was followed by 10 minutes of elongation at 72°C. PCR products were subjected to electrophoresis in 1% agarose gels in Tris-borate-EDTA (ethylenediaminetetraacetic acid) electrophoresis buffer, stained with ethidium bromide, and detected by ultraviolet transillumination. cDNA samples were normalized for the housekeeping gene *GAPDH*.

For real-time PCR, we used a TaqMan Assay-on-Demand gene expression product (Applied Biosystems, Foster City, CA, USA). The expression levels of TIARP, TNFα, and GAPDH (assay ID Mm00475402_m1, Mm00443258_m1, and Mm99999915_g1, respectively; Applied Biosystems) were normalized relative to the expression of GAPDH. Analysis was performed with an ABI Prism 7500 apparatus (Applied Biosystems) under the following conditions: inactivation of possible contaminating amplicons with AmpErase UNG for 2 minutes at 50°C, initial denaturation for 10 minutes at 95°C, followed by 45 thermal cycles of 15 seconds at 95°C and 60 seconds at 60°C. The serum TNFα level was measured by an enzyme-linked immunosorbent assay (ELISA) kit (eBioscience, Inc., San Diego, CA, USA). After conditioning, the detection limit of TNFα concentration was 2 μg/mL.

### Preparation of anti-TIARP and anti-STEAP4 antibodies

One rabbit was immunized subcutaneously by TIARP peptide_5–19 _(HADEFPLTTDSSEKQ, amino-terminal peptide coupled to keyhole limpet hemocyanin) or human ortholog STEAP4 peptide_3–15 _(KTCIDALPLTMNS) [8] with CFA four times, on days 0, 14, 28, and 42. The rabbit was sacrificed on day 52, and serum was collected. Serum was first purified by protein A column and then affinity-purified by TIARP-peptide_5–19 _or STEAP4 peptide_3–15 _column. The purified fraction was confirmed by TIARP peptide_5–19 _or STEAP4 peptide_3–15 _ELISA.

### Western blotting

The cells were washed with PBS and incubated with lysis buffer (pH 7.4, 50 mM Tris-HCl, 5 mM MgCl_2_, 2 mM phenylmethylsulfonyl fluoride [PMSF], and 0.5% NP-40). Where indicated, protein concentrations were quantified using the bicinchoninic acid reagent (Pierce, Rockford, IL, USA). Samples (10 μg of total protein) were separated by SDS-PAGE (4/20% acrylamide; Daiichi Pure Chemicals Co., Ltd., Tokyo, Japan) and transferred to polyvinylidene fluoride membranes (Bio-Rad Laboratories, Inc., Hercules, CA, USA). All subsequent wash buffers contained 0.05% Tween-20 in PBS. Four percent Block Ace (Dainippon Pharmaceutical, Osaka, Japan) was used to block the membranes and to dilute antibodies. Rabbit polyclonal anti-TIARP antibodies and rabbit anti-actin antibodies (Sigma-Aldrich, Munich, Germany) were used at 1:3,000 dilution. Horseradish peroxidase (HRP)-conjugated anti-rabbit secondary antibodies (1:6,000 dilution; Bio-Rad Laboratories, Inc.) were used to visualize bound anti-TIARP antibodies or anti-actin antibodies with the ECL [enhanced chemiluminescence] Western blot detection kit (Amersham, now part of GE Healthcare, Little Chalfont, Buckinghamshire, UK).

### Treatment with anti-tumor necrosis factor-alpha monoclonalantibody

We used commercially available anti-TNFα mAb (eBioscience, Inc.). For a control antibody, we used similar amounts of rat IgG1 isotype control (R&D Systems, Inc., Minneapolis, MN, USA). Just after the onset of arthritis (on day 8), a single dose of 100 μg of anti-TNFα mAb or control antibody was injected. Spleen was harvested at the indicated time points and analyzed for TIARP expression. Three independent experiments were performed.

### Identification of TIARP-positive cells in splenocytes of mice with glucose-6-phosphate isomerase-induced arthritis

The spleens were harvested on day 12 after GPI immunization and single-splenocyte cell suspensions were prepared as described above. CD4^+^, CD19^+^, CD11b^+^, and CD11c^+ ^cells from splenocytes were isolated by magnetic beads using the MACS™ [magnetic-activated cell sorting] system (Miltenyi Biotec, Bergisch Gladbach, Germany). The cells contained more than 97% CD4^+^, CD19^+^, CD11b^+^, and CD11c^+ ^cells as confirmed by fluorescence-activated cell sorting analysis. The cells were dispensed at 1 × 10^6 ^cells to analyze the expression of TIARP mRNA.

### Immunohistochemical staining for TIARP/STEAP4

At the indicated time points, the ankles of the mice were removed, fixed, decalcified, and paraffin-embedded. Sections (5-μm thick) were stained with hematoxylin and eosin and were evaluated for histological changes. For immunohistochemical study, endogenous peroxidase activity was inhibited using 3% hydrogen peroxidase in methanol. Sections were blocked by 5% bovine serum albumin in PBS for 10 minutes and then incubated with rabbit anti-TIARP antibody (1:100 dilution) or normal rabbit Ig (1:100 dilution; Dako, Tokyo, Japan). Isotype-matched HRP-conjugated anti-rabbit IgG antibody (Bio-Rad Laboratories, Inc.) was added for 30 minutes. HRP activity was detected using 3,3-diaminobendine (DAB) (Nichirei Corporation, Tokyo, Japan) as a substrate. The stained sections were counterstained with Mayer's hematoxylin for 10 seconds and mounted with aqueous mounting medium.

For human STEAP4 staining, synovial tissues were obtained after informed consent was given by RA patients at the time of joint replacement. All RA patients satisfied the classification criteria of the American College of Rheumatology (1987) [9]. The synovium was embedded in optimal cutting temperature compound and frozen in dry ice isopentane, and 5-μm-thick sections were mounted at -25°C. Anti-human STEAP4 polyclonal antibody conjugated with fluorescein isothiocyanate (FITC protein labeling kit; Pierce) and purified anti-human CD68 (BD Pharmingen, San Diego, CA, USA) conjugated with rhodamine (1:100 dilution, Rhodamine protein labeling kit; Pierce) were used. Nuclei were counterstained with 4'-6'-diamidine-2-phenylindole dihydrochloride (DAPI) (Molecular Probes, Inc., now part of Invitrogen Corporation, Carlsbad, CA, USA). The stained sections were examined under a fluorescent microscope (model FW4000; Leica Microsystems, Tokyo, Japan).

### Patients and analysis of human peripheral blood mononuclear cells and synovium for STEAP proteins

Peripheral blood mononuclear cells (PBMCs) from three female patients with RA and three healthy control subjects were obtained. All RA patients satisfied the classification criteria of the American College of Rheumatology (1987) [9]. Synovial tissues from 36 RA and 19 osteoarthritis (OA) patients were obtained at the time of total knee replacement. Written informed consent was obtained from all subjects, and the study was approved by the ethics review committee. Total RNA was extracted with ISOGEN in accordance with the protocol provided by the manufacturer. cDNA was obtained by reverse transcription with a commercially available kit. The following primers were used: STEAP2 sense 5'-CCTACAGCCTCTGCTTACCG-3' and antisense 5'-GAGGGCAAAACAAGAGCAAG-3', STEAP3 sense 5'-GCCAGAAGAGATGGACAAGC-3' and antisense 5'-GGTGCTCTTGCTCTGTAGGG-3', STEAP4 sense 5'-GCTCTCCAGTCAGGAGCACT-3' and antisense 5'-CACACAGCACAGCAGACAAA-3', and GAPDH sense 5'-GAAGGTGAAGGTCGGAGTC-3' and antisense 5'-GAAGATGGTGATGGGATTTC-3'. For real-time PCR, we used a TaqMan Assay-on-Demand gene expression product (Applied Biosystems). The expression level of STEAP4 was normalized relative to the expression of GAPDH. Methods were described above.

### Statistical analysis

All data were expressed as mean ± standard error of the mean. Differences between groups were examined for statistical significance using the Mann-Whitney *U *test. A *P *value of less than 0.05 denoted the presence of a statistically significant difference.

## Results

### Induction of glucose-6-phosphate isomerase-induced arthritis

DBA/1 mice were immunized using the human recombinant GPI as reported previously [3,4]. All mice developed arthritis after immunization with 300 μg of GPI. Arthritis was documented at day 8, and severe arthritis was recorded at day 14, with ankle swelling reaching a maximum at day 14 but subsiding gradually on follow-up.

### Overexpression of tumor necrosis factor-induced adipose-related protein in splenocytes of arthritic mice

**Figure 1:**
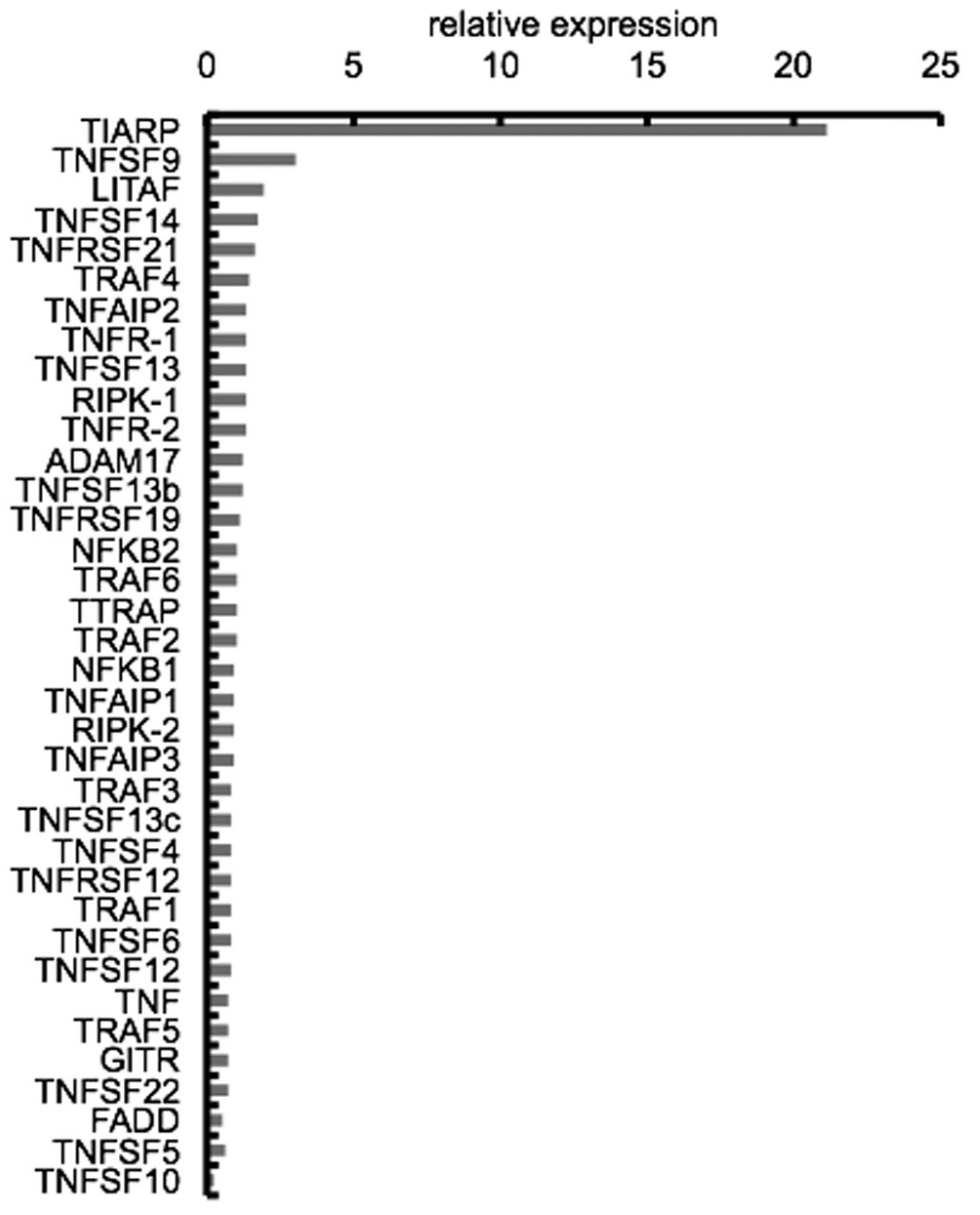
Upregulation of tumor necrosis factor-alpha (TNFα)-related genes in splenocytes of mice with glucose-6-phosphate isomerase (GPI)-induced arthritis. The mRNA expression levels of TNF-related genes in splenocytes of mice with GPI-induced arthritic (at day 10) relative to control splenocytes are shown. TNFα-induced adipose-related protein (TIARP) was specifically and strongly induced in splenocytes. GeneChip analysis was performed by gene expression software. ADAM17, a disintegrin and metallopeptidase domain 17; FADD, Fas (tumor necrosis factor receptor superfamily 6)-associated via death domain; GITR, glucocorticoid-induced tumor necrosis factor-related protein-D mRNA; LITAF, lipopolysaccharide-induced tumor necrosis factor-alpha factor; NFKB1, nuclear factor kappa B subunit p105; NFKB2, nuclear factor kappa B subunit p100; RIPK, receptor (tumor necrosis factor receptor superfamily)-interacting serine-threonine kinase 1 and 2; TNFAIP, tumor necrosis factor alpha-induced protein; TNFR, tumor necrosis factor receptor; TNFRSF, tumor necrosis factor receptor superfamily; TNFRSF12, WSL-1-like protein; TNFRSF22, tumor necrosis factor receptor family member SOBa mRNA; TNFSF, tumor necrosis factor (ligand) superfamily; TRAF, tumor necrosis factor receptor-associated factor; TTRAP, tumor necrosis factor receptor-associated factor and tumor necrosis factor receptor-associated protein.

To explore TNF-related genes in GPI-induced arthritis, we performed GeneChip analysis using arthritic splenocytes and control-immunized splenocytes. Among the arrayed TNFα-related genes, TIARP mRNA was highly expressed in arthritic splenocytes, with levels exceeding more than 20 times those of the control splenocytes (Figure 1). This finding suggests that TIARP protein is an important molecule in TNFα-dependent arthritis. The data discussed in this publication have been deposited in the Gene Expression Omnibus (GEO) of the National Center for Biotechnology Information (Bethesda, MD, USA) and are accessible through GEO Series accession number [GEO:GSE17272] [10].

### Tumor necrosis factor-alpha and TIARP expression in glucose-6-phosphate isomerase-induced arthritis

**Figure 2:**
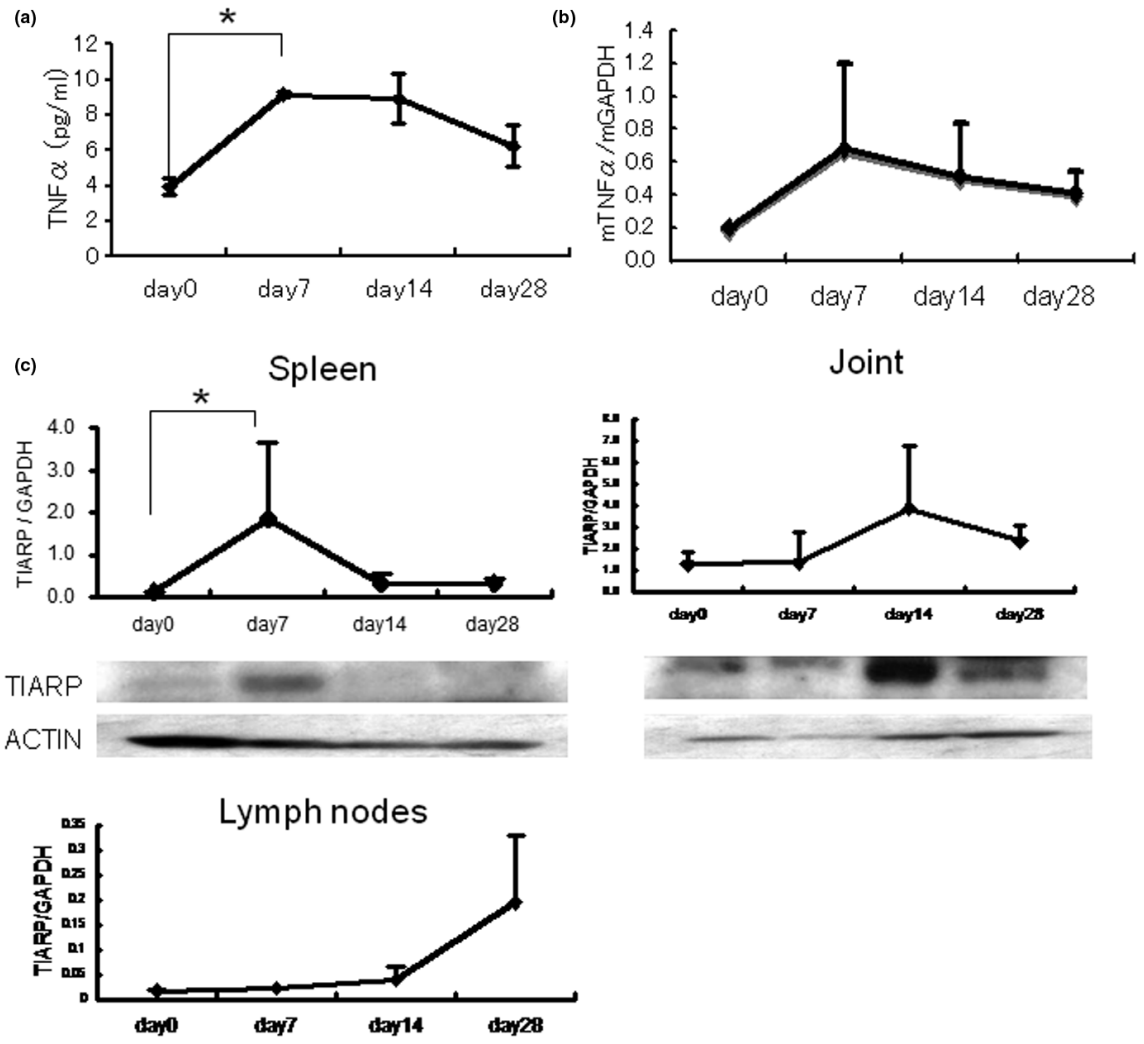
Serial changes in expression levels of tumor necrosis factor-alpha (TNFα) and TIARP in glucose-6-phosphate isomerase (GPI)-induced arthritis. Serial changes in TNFα concentrations in **(a) **serum and **(b) **arthritic joints and **(c) **TIARP mRNA and protein expression in spleens (left and middle panels) and arthritic joints (right panel) by real-time polymerase chain reaction (PCR) and Western blotting in mice with GPI-induced arthritis. As shown in the bottom panel of **(c)**, TIARP mRNA in lymph nodes was also analyzed. Arthritis appeared on days 7 and 8, peaked in severity on day 14, and then gradually subsided. High expression levels of TIARP mRNA and proteins were detected in splenocytes on day 7 (the onset of arthritis). In joints, the expression of TIARP mRNA and protein was correlated with joint swelling (days 14 and 28). Data are mean ± standard error of the mean of five mice per group. *P < 0.05 (Mann-Whitney *U *test). GAPDH, glyceraldehydes-3-phosphate dehydrogenase; mTNFα, murine tumor necrosis factor-alpha; TIARP, tumor necrosis factor alpha-induced adipose-related protein.

To determine the correlation between TNFα and TIARP in GPI-induced arthritis, the time course of TIARP expression was analyzed. Serum TNFα levels were elevated at day 7 (onset of arthritis, *P *< 0.05), were at the same elevated levels at day 14 (peak of arthritis), and then subsided to the basal level at day 28 (Figure 2a). In contrast, the TNFα mRNA expression level in arthritic joints tended to increase at day 7, though insignificantly, in mice with GPI-induced arthritis. The expression level decreased later to basal levels (Figure 2b).

Both real-time PCR and Western blotting showed upregulation of TIARP mRNA and protein expression at day 7 in splenocytes of mice with GPI-induced arthritis (Figure 2c, left panel). In the joints of the same mice, upregulation of TIARP mRNA and protein was noted at days 14 and 28, and the expression correlated with joint swelling (Figure 2c, right panel). Moreover, in lymph nodes, TIARP mRNA was upregulated at day 28. But the expression of TIARP mRNA in lymph nodes was very weak compared with the other tissues (Figure 2c, bottom panel). We also confirmed that the mRNA expression of TIARP in joints was upregulated at day 28, but not at day 14, in mice with collagen-induced arthritis and that expression correlated with joint swelling (data not shown). These findings suggest that the systemic upregulation of TNFα and TIARP is involved in the early phase of the disease and that TIARP expression in arthritic joints seems to correlate with joint swelling.

### Treatment with anti-tumor necrosis factor-alpha monoclonal antibody suppresses TIARP expression

**Figure 3:**
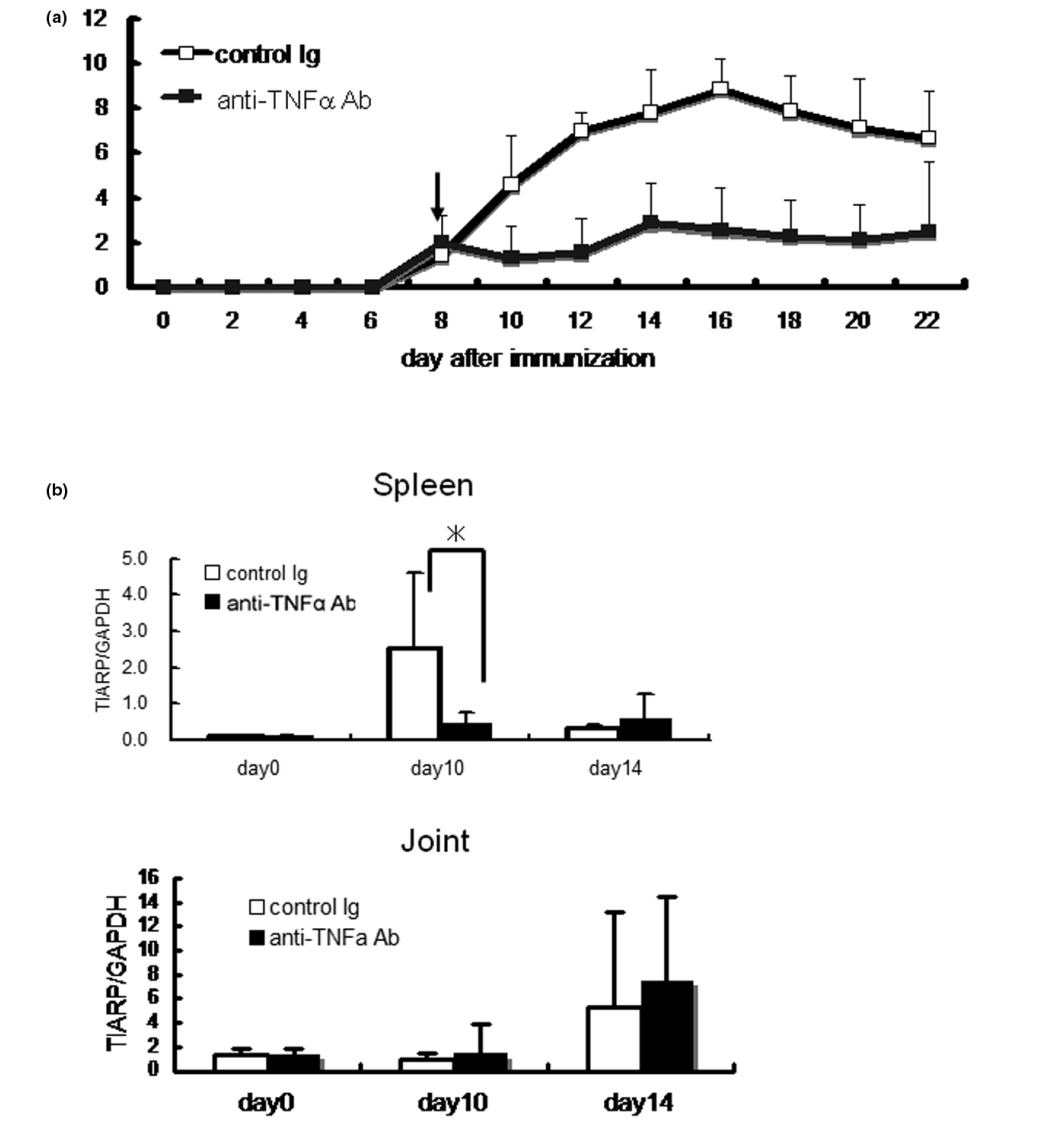
Suppression of TIARP mRNA by treatment with anti-tumor necrosis factor-alpha monoclonal antibody (anti-TNFα mAb). **(a) **The development of arthritis was blocked by administration of anti-TNFα mAb in mice immunized with glucose-6-phosphate isomerase. Data represent arthritis scores. **(b) **In spleen, administration of anti-TNFα mAb suppressed the rise in TIARP mRNA (on day 10) (solid bars), but not control Ig (open bars). However, in joints, expression of TIARP mRNA was almost comparable after the administration of anti-TNFα mAb or control Ig. Data are mean ± standard error of the mean of five mice per group. *P < 0.05 (Mann-Whitney *U *test). GAPDH, glyceraldehydes-3-phosphate dehydrogenase; TIARP, tumor necrosis factor alpha-induced adipose-related protein.

To test the therapeutic efficacy of anti-TNFα mAb, we injected anti-TNFα mAb after clinical onset of arthritis at day 8. A single injection of 100 μg of anti-TNFα mAb at day 8 ameliorated the disease, as indicated by a rapid fall in the semiquantitative score of arthritis (Figure 3a) [3]. To explore the relevance of the therapeutic effect of anti-TNFα mAb on TIARP expression, we evaluated TIARP expression after injection of anti-TNFα mAb in mice with GPI-induced arthritis. Treatment of mice with anti-TNFα mAb resulted in downregulation of TIARP expression in spleen relative to control Ig injection, although no treatment-related change in TIARP expression was noted at day 14 (*P *= 0.03) (Figure 3b, top panel). However, in joints, expression of TIARP mRNA was almost comparable between the treatment with anti-TNFα mAb and control Ig. These results suggest that TNF antagonism induces TIARP downregulation and results in the amelioration of arthritis.

### CD11b^+ ^cells are the main source of TIARP mRNA in splenocytes of arthritic mice

**Figure 4:**
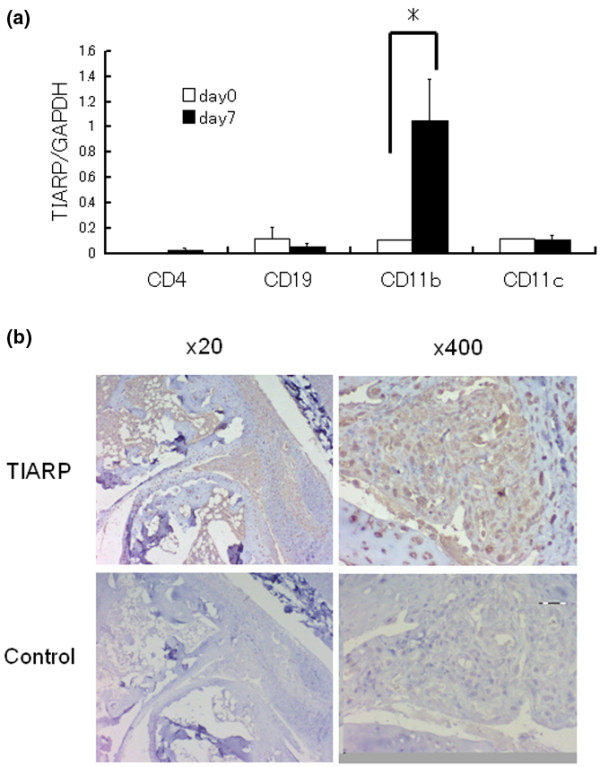
Identification of TIARP-expressing cells in splenocytes and joints of arthritic mice. **(a) **Splenocytes were isolated from naïve (day 0) mice and mice with glucose-6-phosphate isomerase (GPI)-induced arthritis and then were separated into four groups (CD4^+^, CD19^+^, CD11b^+^, and CD11c^+^) by magnetic-activated cell sorting. The expression of TIARP mRNA was analyzed by quantitative real-time polymerase chain reaction at days 0 and 7. TIARP mRNA was expressed mainly on CD11b^+ ^cells in arthritic mice. Data are mean ± standard error of the mean of five mice per group. *P < 0.05 (Mann-Whitney *U *test). **(b) **Joints were obtained from mice with GPI-induced arthritis on day 14 and stained with anti-TIARP antibodies (top panels) and control antibodies (bottom panels). Inflamed synovial tissue of arthritic mice was stained with anti-TIARP antibodies. GAPDH, glyceraldehydes-3-phosphate dehydrogenase; TIARP, tumor necrosis factor alpha-induced adipose-related protein.

In the next set of experiments, splenocytes of arthritic mice were separated into CD4^+^, CD19^+^, CD11b^+^, and CD11c^+ ^cells by MACS. In naïve mice, CD19^+^, CD11b^+^, and CD11c^+ ^cells expressed TIARP, and induction of arthritis was associated with upregulation of TIARP mRNA in CD11b^+ ^cells, as demonstrated by quantitative PCR (*P *< 0.05 at day 7) (Figure 4a). These findings suggest the induction of TIARP in CD11b^+ ^cells in splenocytes of arthritic mice, especially during the early phase of the disease.

### Localization of TIARP protein in proliferative synovium

Next, immunohistochemical analysis was conducted to determine the distribution of TIARP in the arthritic joints. For this purpose, we generated polyclonal anti-TIARP antibodies using rats, as described previously [5]. TIARP protein was clearly identified in the proliferative synovium of arthritic joints of mice (at day 14) (Figure 4b, top panels), whereas almost no signal was detected in naïve mice (Figure 4b, bottom panels). While these findings indicate TIARP protein expression in the synovium, the results do not link such expression with an ameliorative or damaging effect on the synovium.

### Overexpression of STEAP4 in joints of rheumatoid arthritis patients and its localization in CD68^+ ^cells

**Figure 5:**
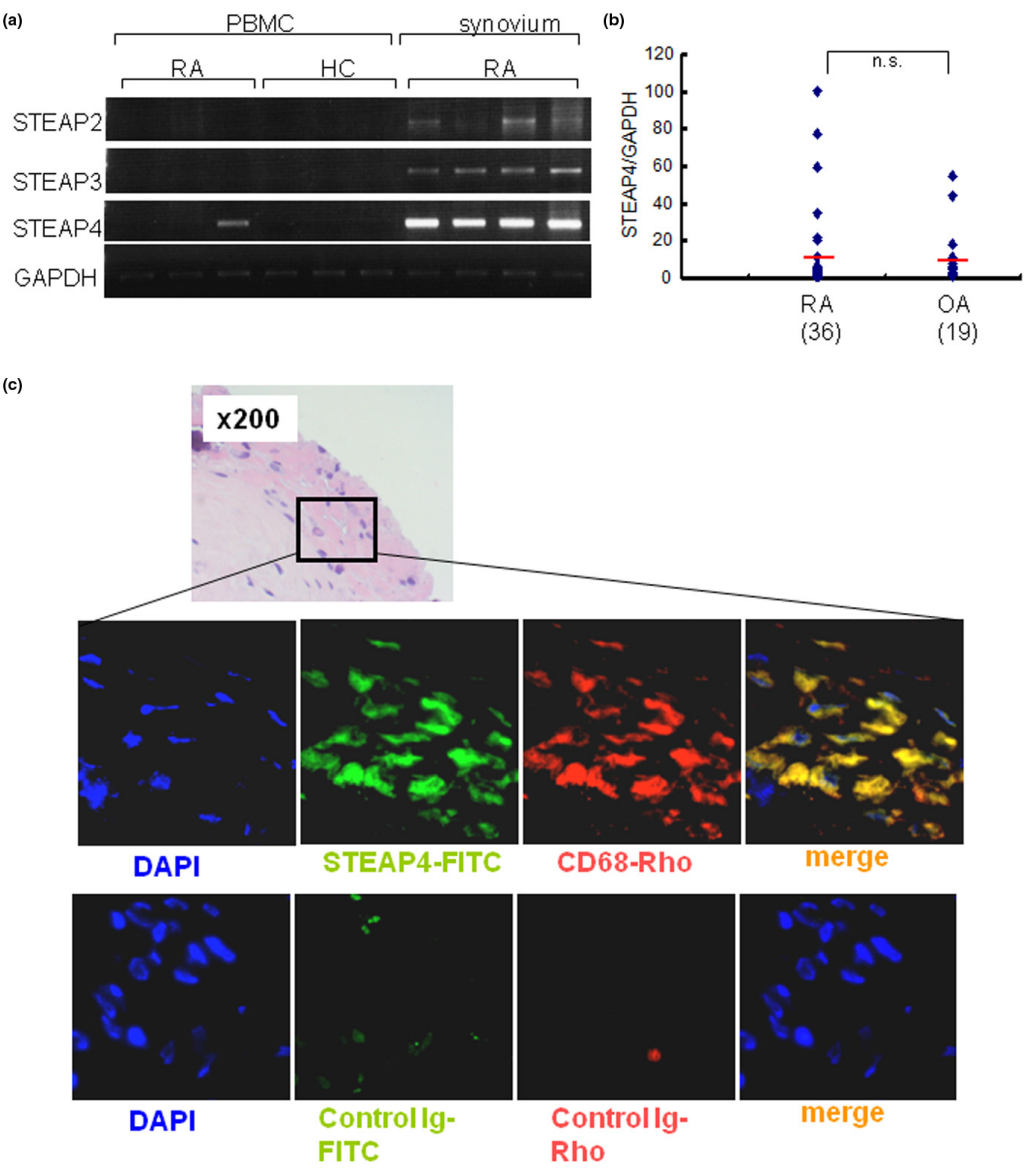
Analysis of STEAP mRNA expression by reverse transcription-polymerase chain reaction (RT-PCR) in peripheral blood mononuclear cells (PBMCs) and synovia of rheumatoid arthritis (RA) patients and healthy subjects (HC) and immunohistochemistry for STEAP4 in RA synovium. **(a) **The expression of STEAP4 mRNA and other family members (STEAP2 and STEAP3 mRNAs) was analyzed in PBMCs (RA and HC) and RA synovium using RT-PCR. In PBMCs, STEAP4 mRNA was detected in a patient with RA (1/3). Surprisingly, STEAP4 mRNA was highly expressed in all four RA synovia whereas only faint staining was noted for other members of the STEAP family. **(b) **The expression of STEAP4 mRNA in synovium with RA and osteoarthritis (OA) patients. STEAP4 mRNA expression was not statistically different between the RA and OA groups. **(c) **Co-localization of STEAP4 and CD68 in RA synovium. Images of immunohistochemistry using 4'-6-diamidino-2-phenylindole (DAPI), fluorescein isothiocyanate (FITC)-anti-STEAP4, and rhodamine-anti-CD68 and a merged image are shown in the middle panels, and images with conjugated control Ig are shown in the bottom panels. Consecutive hematoxylin-and-eosin staining is shown in the top panel. GAPDH, glyceraldehydes-3-phosphate dehydrogenase; n.s., not significant; STEAP, six-transmembrane epithelial antigen of the prostate.

To determine the role of STEAP4 (the human ortholog of mouse TIARP)in human RA, we analyzed PBMCs from RA patients and healthy subjects and synovia from RA patients. For comparison, we also screened other STEAP family members such as STEAP2 and STEAP3 using the same method. For PBMCs, STEAP4 mRNA was detected in only one RA patient (1/3). Importantly, STEAP4 mRNA was highly expressed in all four RA synovia whereas only faint bands were noted for other STEAP families (Figure 5a). Next, using several numbers of synovial tissues from patients with RA and OA, we investigated the expression of STEAP4 mRNA in synovium of patients with RA and OA. Relative expression of STEAP4 was almost comparable between RA and OA, although expression variation tended to be enhanced in RA synovium (Figure 5b). Moreover, immunohistochemical analysis of synovia of RA patients showed co-localization of STEAP4 protein with CD68, a marker for human macrophages (Figure 5c). These findings suggest that STEAP4 is specifically expressed in joints and is localized with CD68^+ ^cells.

## Discussion

Although the therapeutic effect of TNF antagonists is confirmed in RA [1], only a few animal models of arthritis have been used to confirm the beneficial effects of TNF antagonists. For example, a recent study reported the therapeutic effect of anti-TNF mAb in DNaseII, type I interferon receptor (IFN-IR) double-knockout mice [11], although this was not a genetically unaltered mouse. Furthermore, Schubert and colleagues reported the protective effect of TNF antagonist in GPI-induced arthritis [2] and arthritis was clearly B cell-dependent [12]. We recently demonstrated the therapeutic effect of TNF antagonist in GPI-induced mice. Thus, it is important to explore TNF-regulated genes in the latter model to understand the mechanisms of action of TNFα antagonists in RA patients. When the GeneChip analysis was used, the present results showed upregulation of TIARP mRNA in the spleen of arthritic mice. TIARP was first identified as TNFα-induced cell surface protein in adipose tissues and is also known to be localized in the liver, kidney, heart, and skeletal muscle [5]. This protein was detected in the course of adipocyte differentiation and conversion and is also induced by IL-6 [6]. In this study, we confirmed its induction in CD11b^+ ^splenocytes in arthritis and we confirmed that it is upregulated in the arthritic synovium of murine GPI-induced arthritis. These findings suggest the involvement of TIARP in the process of proliferation or differentiation state induced by inflammation. In fact, previous studies indicated that TIARP is induced by TNFα and IL-6 in adipocytes [5,6]. TNFα and IL-6 are pleiotropic cytokines known to play crucial roles in human RA, and significant therapeutic effects of their antagonists have been confirmed in recent years [1,13]. In GPI-induced arthritis, both TNFα and IL-6 antagonists have protective effects [3,4], and these cytokines play important roles in the induction of arthritis in collaboration with autoantibodies (anti-GPI antibodies) [14]. However, there is no clear scenario of balance between IL-6 and TNFα in arthritis. In TIARP knockdown animals, exposure to TNFα induced a greater amount of IL-6, suggesting a crucial role of TIARP in the balance between TNFα and IL-6 [15]. It is possible that TIARP expression plays a downregulatory role in the inflammatory cascade.

At this stage, there is no information on whether TIARP act in an antagonistic or agonistic manner with arthritis. However, one report on STAMP2 (a homolog of TIARP protein) [15] confirmed (a) upregulation of inflammatory cytokines such as TNFα and IL-6 in STAMP2-deficient mice, (b) upregulation of macrophage-specific antigens such as CD68 and CD11b, (c) infiltration of CD68^+ ^cells in adipose tissues, and (d) STAMP2-induced suppression of IL-6 expression upon stimulation by TNFα. These findings suggest that STAMP2 (TIARP) suppresses inflammatory cytokines such as TNFα and IL-6 and also blocks the activation of macrophages/monocytes.

Is this scenario applicable to patients with RA? In humans, the STEAP protein family was identified in prostate tumors [16,17] and is also known to be involved in cell apoptosis [18]. Among this family of genes, STEAP4 is highly expressed in the bone marrow, followed by placenta and fetal liver [19]. The STEAP4 expression was induced by TNFα in human adipose tissue [20] and also by TNFα in human synovial cells (our preliminary result). However, there is no report regarding the expression of this molecule in articular joints. The present study identified the expression of human ortholog STEAP4 in the synovium, especially in CD68^+ ^macrophages of patients with RA. In addition, our preliminary data using human synovial cell lines provide evidence that TNFα stimulation enhances the expression of STEAP4 protein and that a stably expressed form of STEAP4 is partially co-localized with endosomes (Tanaka and colleagues, manuscript in preparation). Further large-scale studies are required to assess the expression of STEAP4 in the joints and PBMCs of RA patients before and after treatment with TNF antagonists.

## Conclusions

The results of the present study highlighted the important role of TIARP/STEAP4, a relatively new TNF-induced protein, in autoimmune arthritis in both mice and humans.

## Abbreviations

CFA: complete Freund's adjuvant; ELISA: enzyme-linked immunosorbent assay; GAPDH: glyceraldehydes-3-phosphate dehydrogenase; GEO: Gene Expression Omnibus; GPI: glucose-6-phosphate isomerase; GST: glutathione S-transferase; HRP: horseradish peroxidase; IL-6: interleukin-6; mAb: monoclonal antibody; MACS: magnetic-activated cell sorting; MW: molecular weight; OA: osteoarthritis; PBMC: peripheral blood mononuclear cell; PBS: phosphate-buffered saline; PCR: polymerase chain reaction; RA: rheumatoid arthritis; STEAP: six-transmembrane epithelial antigen of the prostate; TIARP: tumor necrosis factor alpha-induced adipose-related protein; TNF: tumor necrosis factor; TNFR: tumor necrosis factor receptor.

## Competing interests

The authors declare that they have no competing interests.

## Authors' contributions

AI helped to write the manuscript, conceive of the study, perform all experiments, and coordinate statistical study. IM wrote the manuscript and conceived of the study. YT helped to perform all experiments and coordinate statistical study. KI participated in the clinical assessment. AK and NO collected the synovial samples. DG and SI participated in discussion. TS participated in the full design and coordination of the study. All authors read and approved the final manuscript.
